# Computations that sustain neural feature selectivity across processing stages

**DOI:** 10.1371/journal.pcbi.1013075

**Published:** 2025-06-20

**Authors:** Ryan J. Rowekamp, Tatyana O. Sharpee

**Affiliations:** 1 Computational Neurobiology Laboratory, The Salk Institute for Biological Studies, La Jolla, California, United States of America; 2 Department of Physics, University of California - San Diego, La Jolla, California, United States of America; Centre National de la Recherche Scientifique, FRANCE

## Abstract

Biological visual systems are celebrated for their ability to reliably and precisely recognize objects. However, the specific neural mechanisms responsible for this capability remain largely elusive. In this study, we investigate neural responses in the visual areas V1, V2, and V4 of the brain to natural stimuli using a framework that includes quadratic computations in order to capture local recurrent interactions, both excitatory and suppressive. We find that these quadratic computations and specific coordination between their elements strongly increase both the predictive power of the model and the neural selectivity to natural stimuli. Particularly important were (i) coordination between excitatory and suppressive features to represent mutually exclusive hypotheses regarding incoming stimuli, such as orthogonal orientations or opposing motion directions in area V4, (ii) balance in the contribution of excitatory and suppressive components and its maintenance at similar levels across stages of processing, and (iii) refinement of feature selectivity between stages, with earlier stages representing broader category of inputs. Overall, this work describes how the brain could use multiple nonlinear mechanisms to increase selectivity of neural responses to natural stimuli.

## Summary

Although machine vision has improved greatly, it differs from human vision in many respects. One of the crucial ways in which it is different is that machine vision can be tricked by adding tiny perturbations that are imperceptible to a human. To understand how biological vision achieves its robustness, we analyzed responses of neurons from several brain areas performing visual recognition. We found that these responses could be better described when standard machine vision models were modified to include quadratic computation into their core element. Two properties of this quadratic computation increased neuronal selectivity. First, quadratic computation included suppressive elements that suppressed responses to visual patterns that would be incongruent with the main pattern. Second, suppressive and excitatory patterns were balanced in their number, size, and other properties in almost all brain regions studied. The results highlight unique features of brain computation that uphold neural selectivity throughout different processing stages.

## Introduction

**Fig 1:**
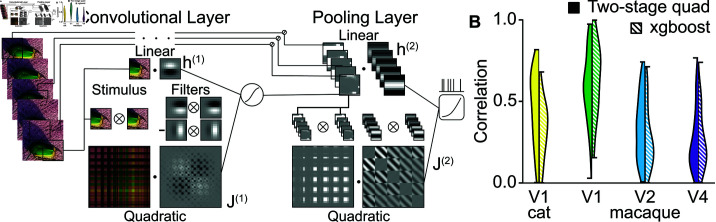
Two-layer quadratic convolutional model accounts for neural responses to natural stimuli across in four brain regions. **A.** Schematic of the model. For the first, convolutional layer, patches from different positions and times from the stimulus (left) are passed through identical quadratic subunits. The subunits contain a linear filter and a quadratic filter (middle). The linear filter operates like a typical neural network subunit. The quadratic filter operates on the outer product of the input, allowing sensitivity to multiple features in the input. The outputs of the linear and quadratic filters are added together along with a scalar bias and passed through a sigmoid that thresholds and saturates the response. The responses from each position and time form the outputs of the first layer. These responses are pooled by another linear and quadratic filter (right). Their outputs are added with another bias and rectified with a softplus function to produce a predicted firing rate. **B.** Comparison with machine learning models. The quadratic convolutional model has comparable performance with XGBoost, a gradient-boosted decision tree model focused only on predicting responses that is not amenable to interpretation of its parameters (mean ±s.e.m. for cat V1 values were 0.45±0.03 for the quadratic convolutional and 0.32±0.03 for xgboost. In primate V1: 0.59±0.03 for the quadratic convolutional model versus 0.63±0.03, V2: 0.34±0.02 versus 0.28±0.02, V4: 0.259±0.015 versus 0.259±0.014).

The visual system takes a pattern of photons interacting with rod and cone cells in the retina and processes this pattern through multiple stages until it is able recognize the presence of various objects within the scene. The broad strokes of this process are known (finding points of contrast, extracting edges from these points, combining these edges into curves and intersections, etc.), but the details of how the presence of secondary features modifies the responses of neurons is less well understood. To address this goal, we have designed a computational model that is scalable and can be used to analyze neural responses across different stages of visual processing. The model extends the standard convolutional neural network model to include nonlinearities designed to mimic properties of real neural networks, including local recurrent interactions, and at the same time remains computationally tractable. Specifically, the model adds a quadratic filter in addition to the standard linear filter in the basic neuron-like subunit of the model. A version of this model that included the quadratic filter only in the first layer was previously used to characterize responses of neurons within the visual area V2 [1]. Applying this model to responses of neurons from the next stage of processing, the visual area V4, we found that it could not adequately account for these neural responses. Therefore, we further extended the model to include a quadratic filter in all layers, not just the first convolutional layer (Fig 1A). From a computational perspective, the quadratic filter provides an effective tool for capturing the contributions of local recurrent circuitry at each stage of processing [2]. It can also describe multiple overlapping visual features that may affect neural responses locally [3,4]. In particular, this allows one to take into account the suppressive features that have been shown to be important for enhancing robustness of neural responses at the primary [3,5–9] and the secondary [10–12] visual areas V1 and V2. Because all layers of the model have a quadratic filter, the framework opens the possibility for discovering the general rules for suppressive interactions.

Our model (shown schematically in Fig 1A) is based on the convolutional neural networks often used in computer vision [13]. In these models, each layer is composed of subunits whose activity is determined by a multi-dimensional linear combination of their local inputs that is then passed through a nonlinear function. The nonlinear function thresholds the response and (depending on the form of the function) may cause the response to saturate at some maximum value. The outputs of these subunits are convolved across the stimulus to provide a response at each location in the image that is then used as the input for the next layer. The advantage of convolutional models is that they provide an efficient means of encoding positional invariance which enables recognition of objects regardless of where they appear in the visual field.

We build upon this base model by adding a multidimensional quadratic filter in addition to the linear filter at each layer of the model. This is motivated in a couple of ways. First, this may be thought of as adding the quadratic term of a Taylor expansion of the nonlinear function describing the computations performed by upstream neurons as well as the neuron’s own nonlinear processes. The quadratic filter can represent the interactions of multiple features together in contrast to the single feature of the linear component. Second, certain neural responses, such as the phase-invariant response of complex cells in area V1, can be emulated using quadratic terms in what is called an energy model [14].

We use this model to characterize neural responses across different stages of visual processing [15–17], using data from the primary visual cortex (V1) of the monkey [18] and the cat [19], the secondary visual area V2 of the monkey [18], and finally the area V4 of the monkey [20]. In all cases, neural responses were probed with natural stimuli (although there were differences in temporal statistics between different sets of stimuli, discussed below). Data from the cat were obtained under anesthesia, while all primate data were obtained from awake animals [18–20]. For each neuron, the fitting procedure adjusted the rank of the quadratic filter at each layer and the relative size of the convolution layer’s filter to the size of the stimulus. Despite these adjustments, the model remained computationally tractable because it uses at its core the quadratic computational element which by itself allows for convex optimization devoid of local optima [2]. We note that the linear combination is not constrained during optimization, i.e. can be an arbitrary spatiotemporal profile, although after the fit, the resulting profile will be approximated as a combination of Gabor functions.

## Results

### Quadratic convolutional model matches the performance of non-interpretable benchmark model

Before analyzing the structures of our models to infer what computations are being performed by the neurons, we first established that our models were replicating a reasonable fraction of the neuron’s responses. In order to estimate how much of the neuron’s responses can be reasonably replicated, we turned to XGBoost as a benchmark [21]. This algorithm combines the weak predictions from multiple decision trees into a larger model that is far more accurate than its individual parts. Although the XGBoost’s algorithm of forest of decision trees does not lend itself to inspection of how selectivity was achieved, it can give us an estimate of what level of performance we can reasonably expect from more interpretable models. The quadratic convolutional model achieved comparable and, in some cases, substantially better performance than XGBoost (Fig 1B). In the case of V1 neurons, the quadratic convolutional models actually outperformed the benchmark model, and they reached >97% of the benchmark’s performance for higher visual processing stages. The high predictive power achieved by our quadratic convolution model when accounting for neural responses in V1, V2 and V4 validates its use as tool for characterizing the computations performed within these areas.

### Bimodal distribution in the relative dominance of linear and quadratic computations

**Fig 2:**
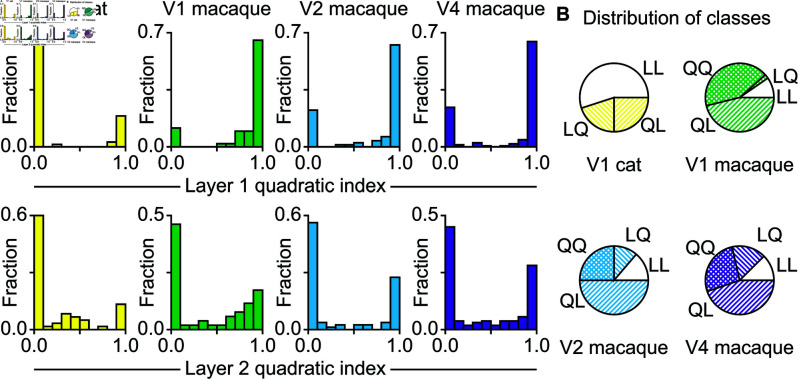
Fraction of variance contributed by quadratic component of first and second layers. **A.** The quadratic index is the variance of the contribution of the quadratic component of a layer divided by the sum of the variances of the outputs of the linear and quadratic filters. Across all areas, most neurons were dominated by either the linear or the quadratic component for both stages, so we divided them into four classes based on which component contributed more to the outputs of the layers. **B.** The distribution of the neurons into four classes: linear in the first layer and linear in the second (LL), linear-quadratic (LQ), quadratic-linear (QL), and quadratic-quadratic (QQ).

Although all neurons were modeled with quadratic filters in both layers, the models did not necessarily need to use the additional quadratic filter for their predictions of the neuron’s responses. In order to determine which parts were relevant to the model’s response and therefore might contain information about the computations performed by the neurons, we calculated a quadratic index, defined as the variance of the output of the quadratic term divided by the sum of the variances of the quadratic term and the linear term. When this index is close to zero, the output of the linear filter dominates the response, and the output of the quadratic filter dominates the response when the index is close to one. We calculated this index for each layer and found that the distribution was strongly bimodal in all cases (Fig 2A). We note that the strength of bimodality is partially dependent on the choice of the sigmoid activation function in the first layer because the strength of bimodality was reduced when the activation function was changed to a rectified linear (RELU) function (S12 Fig). The enhancement of bimodality from the use of sigmoidal nonlinearity is expected because this nonlinearity serves as a classifier. In principle, it can create bimodal distributions even from a purely unimodal distribution [22]. The fact that bimodality persists when model structure is modified to use a more linear function points to an underlying biological mechanism that separates cells into classes based on the strength of the quadratic index at each layer of the model. We consider the following four classes: Linear-Linear (LL), Linear-Quadratic (LQ), Quadratic-Linear (QL), and Quadratic-Quadratic (QQ). This classification builds upon and expands the simple and complex cells definitions in area V1 where cells are classified based on the extent to which they respond to their inputs linearly or nonlinearly [23,24]. The distributions of neurons into these categories for each dataset are shown in Fig 2B.

The quadratic computations were more common in the primate neurons than in the cat neurons. Neurons with quadratic computations in the first stage of the model comprised 3/4 of the data in area V2 and V4, and a similar but even greater fraction among primate V1 neurons. By comparison, QQ neurons were absent in the cat V1 altogether, with QL neurons comprising 1/4 of the population (Fig 2). Comparing the primate and cat V1, the average number of features at the first stage was not statistically different when counting all cells (both quadratic and linear at the first stage, with linear cells counted as one feature). However, looking at only the quadratic neurons in the first stage, these neurons were less frequent in the cat and had more features than quadratic-at-the-first-stage neurons in the primate (S4A and S4B Fig).

Quadratic computations were also important at the second stage, providing a dominant contribution in just under half of neurons in primate V1, V2, V4, with no significant differences between areas, cf. S4 Fig. In the cat, quadratic neurons represented only 1/4 of the V1 population. Comparing neurons that were quadratic-at-the-second-stage, we found that these neurons had similar number of features across all brain regions studied (no statistically significant differences, S4D Fig, ANOVA, F[3,129]=1.69, *p* > 0.05). Because quadratic cells were less common in cat V1, overall across all neurons, the number of features was less in cat V1 compared to V1/V2/V4 of the primate (S4C Fig, ANOVA, F[3,349]=4.06, *p* < 0.01; t-test, cat V1 vs macaque V1, D(110)=4.29, *p* < 0.001; vs macaque V2, D(138)=−2.80, *p*<0.01; vs macaque V4, D(219)=−3.17, *p* < 0.01). Overall, these results highlight the importance of quadratic computations for multi-stage visual processing.

### Cross-orientation suppression between local features

We now describe the local feature selectivity performed by the first layer of the model. The quadratic filter can encode combinations of arbitrary features, so we must use some framework to systematically understand what features the local subunit responds to. The commonly used eigenvector decomposition requires the eigenvectors to be orthogonal [4,25,26], which causes them to become linear combinations of the likely non-orthogonal features that modulate a neuron’s response.

In order to be able to better interpret identified computations in terms of underlying neural circuits, we sought to characterize these features as combinations of edge-detectors, namely four different sets of features based on Gabor wavelets [27]. The first was a set of standard Gabor wavelets. The second were quadrature pairs of wavelets with identical parameters except for a $90^{\circ }$ offset in their phase. Combined quadratically, these pairs could provide phase-invariant response to stimuli of their preferred orientation and wavelength. The third and fourth sets of features were curved versions of the previous sets. Using the AIC criterion, we determined that the curved, non-paired Gabors best fit the quadratic term and used it for the following analysis (S1 Fig).

**Fig 3:**
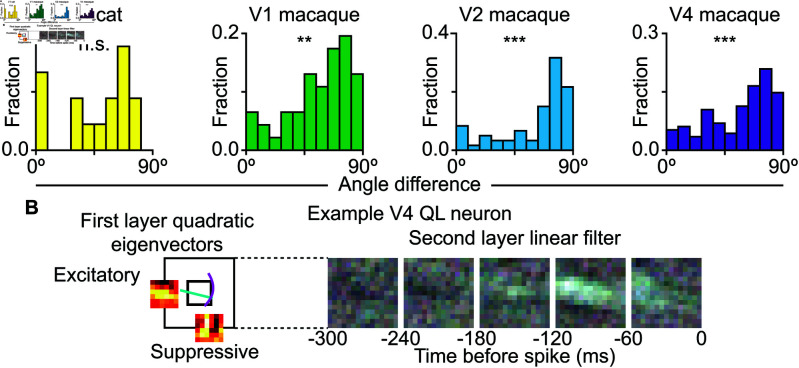
Excitation and suppression are orthogonal to each other in first layer quadratic cells. **A.** Difference between the mean orientation of the excitatory features and the mean orientation of suppressive features at the position of maximum interaction. The distribution is biased towards orthogonality for macaque V1, V2, and V4 but not cat V1. **B.** An example QL neuron from V4 showing the horizontal preference of the excitatory feature and the vertical preference for the suppressive feature. The inner square is the size of the first layer features, and the lines show the peak of the curved Gabors extended out to one σ with the horizontal excitatory feature in cyan and the vertical suppressive feature in magenta. Note that they are orthogonal to each other where they intersect.

A key advantage of using a quadratic filter is that it makes it possible to simultaneously estimate excitatory and suppressive features that affect neural responses at each layer of the model. Considering coordination between excitatory and suppressive features we find that in all primate visual areas, from V1 to V4, excitatory and suppressive features had a strong preference for orthogonal orientation at the point of maximum feature strength (Fig 3A). The bias towards $90^{\circ }$ was significant in all primate areas (Kolmogorov-Smirnov test versus uniform distribution; V1 D(46)=0.26, *p* < 0.01; V2 D(60)=0.39, *p*<0.001; V4 D(115)=0.24, *p* < 0.001). It was not significant in cat V1, likely due to the small number of neurons with a strong quadratic computation at the first layer (D(15)=0.16, *p* > 0.05). Fig 3B shows an example neuron with its orthogonal excitatory and suppressive features as well as how the first layer’s subunits are pooled by the second layer.

Looking more closely at the properties of the fitted Gabors, we found that the orientations of excitatory or suppressive Gabors tended to be aligned with each other, with the mean weighted angular standard deviation being: $5.4^{\circ }$ for cat V1 excitatory features, $5.8^{\circ }$ for cat V1 suppressive features, $7.0^{\circ }$ for macaque V1 excitatory features, $4.8^{\circ }$ for macaque V1 suppressive features, $4.9^{\circ }$ for macaque V2 excitatory features, $5.5^{\circ }$ for macaque V2 suppressive features, $8.4^{\circ }$ for macaque V4 excitatory features, and $5.8^{\circ }$ for macaque V4 suppressive features. Of the four datasets, only the macaque V4 had significantly larger variation in preferred orientation of excitatory features compared to other brain regions (paired difference t-test: cat V1, D(14)=0.15, *p* > 0.05; macaque V1, D(45)=1.41, *p* > 0.05; macaque V2, D(59)=0.51, *p* > 0.05; and macaque V4, D(114)=4.13, *p* < 0.001). The weighted mean of σ was only significantly larger for excitatory features than suppressive features for macaque V4 (paired difference t-test: cat V1, D(14)=1.10, *p* > 0.05; macaque V1, D(45)=1.24, *p* > 0.05; macaque V2, D(59)=1.10, *p* > 0.05; and macaque V4, D(114)=2.34, *p* < 0.001). However, the overall spatial coverage of the combined Gabor features was not significantly different between excitatory and suppressive features for any area (paired difference t-test: cat V1, D(14)=1.64, *p* > 0.05; macaque V1, D(45)=1.69, *p* > 0.05; macaque V2, D(59)=1.37, *p* < 0.05; and macaque V4, D(114)=1.83, *p* > 0.05).

### Orientation alignment across layers

**Fig 4:**
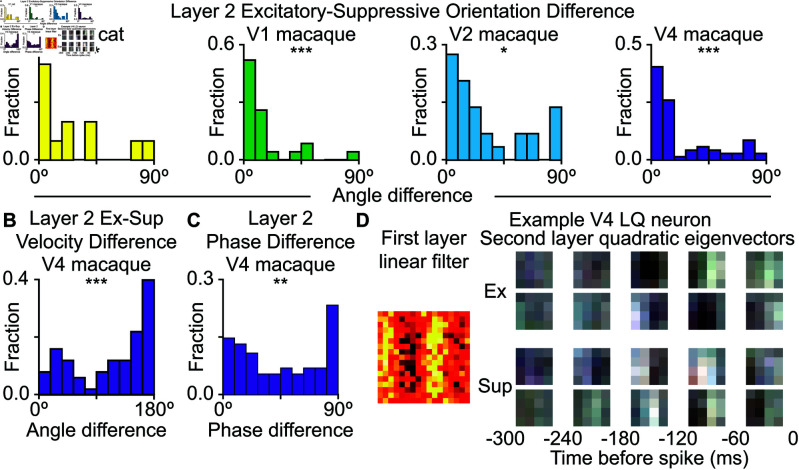
Orientation selectivity aligned between first and second layers. **A.** The difference in the dominant orientation of the first and second layers across V1, V2, and V4. The difference was significantly non-uniform across all three areas (cat V1 D(60)=0.57, $p<3\times 10^{-19}$; macaque V1 D(52)=0.27, $p<8\times 10^{-4}$; macaque V2 D(80)=0.22, $p<9\times 10^{-4}$; macaque V4 D(161)=0.19, $p<2\times 10^{-5}$). **B.** An example cat V1 LL neuron with vertical orientation preference in both its first- and second-layer features. **C.** A schematic showing how multiple edge-detecting features can be pooled along their preferred orientation in order to be combined to select for longer edges. **D.** A frame from the stimulus that provokes the highest response from the model for this neuron. The diagonal line in the stimulus extends beyond the size of the edge-detecting feature in the first layer linear filter. The extension of the feature along the direction of pooling causes a higher response.

Next, we examine the coordination between the convolutional and pooling layers of the model. Using the Fourier transform to find the preferred feature orientation of the dominant filters, we find that the orientations align between layers (Fig 4A). In other words, the dominant feature of the second-layer’s pooling filter has the same primary orientation as the local features it integrates, selecting for continuations of the features preferred by the first layer. This alignment of preferred orientations between convolutional and pooling layers was significant in all areas (Kolmogorov-Smirnov test versus uniform distribution; cat V1: D(60)=0.57, *p* < 0.05; macaque V1: D(52)=0.27, *p* < 0.001; macaque V2: D(80)=0.22, *p* < 0.001; macaque V4: D(161)=0.19, *p* < 0.001). This analysis included all neurons by analyzing the linear or quadratic filter of each layer depending on which was dominant. This selectivity for continuations of the first layers preferred feature can be seen in the stimulus that had the highest predicted firing rate for an example neuron (Fig 4D). In this example, the first layer features describe selectivity for $135^{\circ }$ edge Fig 4B. The second pooling layer also has similar selectivity, which in the case of pooling signifies preference for the set of edges that are shifted in this direction (Fig 4C). The end result is selectivity for a long thin contour that is assembled from constituent pieces. This way of creating selectivity for contours can afford greater robustness than building a single filter with a desired profile. We will test the impact of this arrangement on robustness of neural responses further below.

### Opponent selectivity to motion in area V4

**Fig 5:**
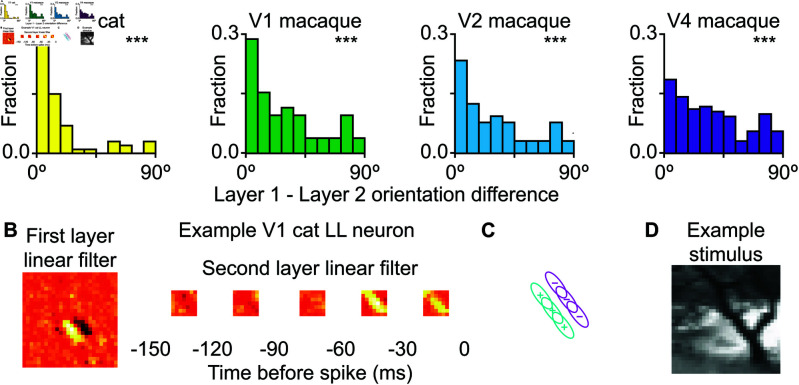
Excitation and suppression are aligned within the second-layer quadratic component. **A.** The dominant orientation of the excitatory and suppressive components of the second layer quadratic cells were aligned for cat V1, macaque V1, V2, and V4. **B.** In macaque V4, second layer quadratic cells excitation and suppression preferred motion of opposite directions. This was not significant for other brain regions. **C.** For macaque V4 cells quadratic in the second layer, there was a preference for orthogonal phases between the first and second excitatory or suppressive features when there were two or more significant dimensions. This combination of features could provide excitation or suppression to motion that is invariant to the position of the object as it crosses the receptive field. **D.** An example LQ neuron from V4 showing the vertical selectivity in both the excitatory and suppressive feature of the second layer. The top two excitatory and suppressive features are shown, with each row showing a separate feature.

Suppressive features were just as prominent at the pooling layer as they were at the first, local layer of the model (S5 Fig). Unlike the cross-orientation suppression that was dominant between local features, here at the pooling layer, for most neurons the excitatory and suppressive features had similar orientations (Fig 5A; Kolmogorov-Smirnov test versus uniform distribution; cat V1: D(12)=0.39, *p* < 0.05, macaque V1: D(23)=0.64, *p* < 0.001; macaque V2: D(29)=0.38, *p* < 0.001; macaque V4: D(69)=0.47, *p* < 0.001). By construction, the excitatory and suppressive features cannot completely overlap with each other, so the excitatory and suppressive features must differ in other stimulus parameters, such as spatial frequency and/or temporal features such as motion. We did not find systemic differences across the population between the excitatory and suppressive features in earlier brain regions (V1 and V2). However, in area V4 the excitatory and suppressive features were systematically organized along opposing directions of motion (Fig 5B, Kolmogorov-Smirnov test versus uniform; cat V1: D(12)=0.18, *p* < 0.05; macaque V1: D(23)=0.14, *p* > 0.05; macaque V2: D(29)=0.18, *p* > 0.05; macaque V4: D(69)=0.28, *p* < 0.001). At this point, it is useful to note that selectivity to motion was stronger in area V4 compared to earlier areas V1 and V2. This was true both for neurons that had predominantly linear or quadratic second layer (S2 Fig).

We tested whether these differences could be due to the differences in the stimuli used in the experiments. The macaque V4 and cat V1 datasets used a natural movie stimulus which contained the temporal correlations of natural motion. The macaque V2 dataset used natural image sequences which didn’t have correlated motion across multiple frames. The macaque V1 dataset used images with simulated saccades, which had limited motion and significantly less power at non-zero temporal frequencies compared to natural stimulus used in V4 and cat V1. By comparison, the flashed natural image sequence dataset had constant power across temporal frequencies, significantly larger than in the natural motion stimulus dataset, cf. S10A Fig. To test whether these differences in stimulus statistics could affect estimation results we generated model spikes from each stimulus set based on the direction selective example neuron. We were able to recover the direction selectivity based on spikes generated from natural movie stimuli, cf. S10B Fig. The spikes generated from flashed scenes (macaque V2 dataset) did not permit us to recover direction selectivity, but we were able to recover a stationary feature. In the case of the simulated saccade stimulus (used in macaque V1 recordings), we were likewise not able to recover temporal modulation. Thus, the observation of motion selectivity in primate area V4 and lack of this observation in primate areas V1 and V2 could in part be due to reduced temporal correlation in the stimulus datasets used in primate V1 and V2 recordings.

In area V4, we were also able to detect phase alignment within excitatory and suppressive features in cases where there were at least two significant excitatory or suppressive dimensions (Fig 5C; Kolmogorov-Smirnov test versus uniform distribution; cat V1: D(24)=0.12, *p* > 0.05; macaque V1: D(44)=0.11, *p* > 0.05; macaque V2: D(58)=0.17, *p* > 0.05; macaque V4: D(128)=0.14, *p* < 0.01). This alignment could provide an invariant excitation to an object moving in the preferred direction regardless of position in the visual field or a corresponding invariant suppression of the response to motion in the opposite direction. We tested this by finding the moving sine gratings that maximized and minimized the response of the example neuron shown in Fig 5D. As expected from the features shown, the model had a maximum response to gratings moving up and to the right and minimum responses moving in the opposite direction (down and to the left) (S11 Fig). The phase of these gratings had little effect on the response, varying by 0.03 for the maximum grating and 0.009 for the minimum grating (standard deviation divided by the mean).

Using the same procedure to find a moving sine grating that maximized the response of each neuron, we then rotated the orientation of each grating and used the directional circular variance to measure how selective the neuron was for its preferred direction (S13 Fig) [28]. The mean circular variance was 0.25±0.03 for cat V1, 0.101±0.013 for macaque V1, 0.115±0.015 for macaque V2, and 0.117±0.013 for macaque V4.

### Coordination between features increases image selectivity

We now examine how the computational motifs described above increase selectivity of neural responses to natural stimuli as defined by the sparseness of the responses: $\text{var}(r)/\text{mean}(r{)}^{2}$.

This quantity is zero when the responses are completely uniform and is maximized when only a single stimulus causes a response. We used sparseness instead of other metrics such as dynamic range because it takes into account all of the response values, instead of just its minimum and maximum values. Sparseness also has analogies to Shannon mutual information, with a maximum sparseness indicating a single, highly informative response to a particular input and sparseness of zero corresponding to a constant output transmitting zero information. This is monotonically related to the measure of sparseness used by Willmore et. al. [29], but it is not bounded on the upper end by 1.

**Fig 6:**
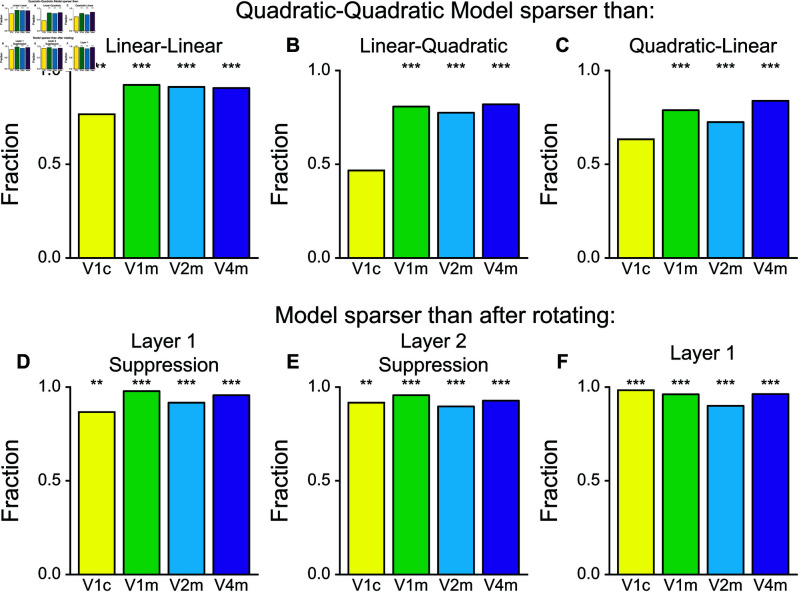
Quadratic computations increase neuronal selectivity to natural stimuli. Alignment between first and second layer, orthogonality of excitation and suppression in the first layer, and alignment between excitation and suppression in the second layer all increase selectivity. For all panels, the bars show the fraction of cells where the responses of the Quadratic-Quadratic model were sparser than the responses of the alternative model. **A-C.** Quadratic-Quadratic model responses were sparser significantly more often than the Linear-Linear, Linear-Quadratic, and Quadratic-Linear alternatives except for cat V1 for the Linear-Quadratic models and Quadratic-Linear models. **D.** For neurons that were quadratic in the first layer, rotating the suppressive features by $90^{\circ }$ reduced the sparseness of the modeled responses. **E.** Rotating the suppressive component of the second layer had the same effect on neurons that were quadratic in that stage. **F.** For all neurons, breaking the alignment between the first and second layer by rotating the first layer reduced the sparseness of the responses. V1c = cat V1. V1m = macaque V1. V2m = macaque V2. V4m = macaque V4.

First, the overall presence of the quadratic filter increased the selectivity of neural responses (Fig 6A-C). For both layers, models that were fit to neural responses without quadratic component produced less sparse responses compared to models where quadratic computations were allowed. This was true in all brain regions, see below for statistical tests, except for cat V1 where quadratic cells constituted a small fraction (binomial test, the full model versus the model without quadratic component at both layer: cat V1 *p* < 0.001, macaque V1 *p* < 0.001, macaque V2 *p* < 0.001, macaque V4 *p* < 0.001; the full model versus the model without a quadratic component in the first layer: cat V1 *p* > 0.05, macaque V1 *p* < 0.001, macaque V2 *p* < 0.001, macaque V4 *p*<0.001; the full model versus the model without quadratic component in the second layer: cat V1 *p* > 0.05, macaque V1 *p* < 0.001, macaque V2 *p* < 0.001, macaque V4 *p* < 0.001). Therefore, in all primate areas, adding a quadratic component to either or both the first and second layer significantly increased the selectivity of neural responses to natural stimuli. This finding is not necessarily expected. For example, a counterexample is provided by complex cells that have stronger quadratic components than simple cells, yet are less sparse than simple cells [30,31].

Furthermore, the three computational motifs observed above (cross-orientation suppression in the convolutional layer, pooling along the preferred direction of the first layer, and orientation alignment between spatiotemporal excitatory and suppressive features in the second layer) all increase the selectivity of the response. Applying a $90^{\circ }$ rotation to the suppressive features of the quadratic filter in the first layer (breaking the orthogonal relationship with the excitatory features) decreased the selectivity of the responses (Fig 6D, binomial test, cat V1 (*p* < 0.01), macaque V1 (*p* < 0.001), macaque V2 (*p*<0.001), and macaque V4 (*p* < 0.001)). Similarly, rotating the suppression within the quadratic filter of the second layer significantly reduced selectivity in all brain regions (*p* < 0.01 for cat V1, *p* < 0.001 for macaque V1, *p* < 0.001 macaque V2, and *p*<0.001 for macaque V4, cf. Fig 6E). We note that there are multiple possibilities where such changes in sparseness would not be observed. For example, were excitatory and suppressive filters were oriented randomly relative to each other, all of these manipulations would not have resulted in statistically significant differences in the sparseness.

Finally, the alignment of features across layers was also important for maintaining response selectivity (Fig 6F). Rotating the first layer relative to the second layer decreased the sparseness in all areas (binomial test versus 0.5, cat V1: *p* < 0.001, macaque V1: *p* < 0.001, macaque V2: *p* < 0.001, and macaque V4: *p* < 0.001). One way this alignment can increase selectivity is by selecting for extensions of elongated contours along their preferred direction. An example of such refinement in selectivity is shown Fig 4D. It is also worth noting that because of correlations present in natural scenes across scales, convolution pre-filtering of visual scenes at the first layer strongly biases the power spectrum of signals received by the second layer (S3 Fig). By aligning with the preferred orientation of the first layer, the second layer is most able to refine the stimuli into different groups and become more selective in its response. This impact on sparseness is perhaps least expected, because it needs to overcome a decrease in sparseness that occurs with standard pooling across spatial positions of similarly tuned sub-units.

## Discussion

We have analyzed neural responses across several stages of visual processing in order to determine computational motifs that increase neuronal selectivity to natural inputs and sustain it across processing stages. To achieve good predictive power, we extended the standard convolutional architecture with a quadratic filter in each layer of the models. This not only increased predictive power, but also revealed specific properties that maintained the neural selectivity to natural scenes.

The quadratic filters included both excitatory and suppressive features that were organized to represent mutually exclusive interpretations of visual inputs. For example, at the first, local layer of the models, excitatory and suppressive features had orthogonal orientation. This finding is consistent with previous analyses of neurons in area V1 and V2 demonstrating that cross-orientation suppression between local features increase the selectivity of these neurons to natural scenes [1,5–9]. Here we extend this finding to other visual areas as well as to other forms of suppressive mechanisms. For example, we find that in area V4, quadratic features at the second layer of the model consist of excitatory and suppressive pairs of features that are selective for opposing directions of motion. This is interesting because area V4 is not conventionally thought of as motion-selective area [32,33]. Nevertheless, motion signals are known to aid in object segmentation [33] and that some motion selectivity was detected in V4 neurons by prior studies [34,35]. We note that while we did not detect as strong motion selectivity in primate areas V1 and V2, this lack of detection was likely due to the diminished motion energy in the stimuli used in those experiments compared to the more natural stimulus used in V4.

The excitatory and suppressive features were balanced in many respects, including their number, spatial extent, and range of orientations within the set of excitatory and suppressive features for each neuron. This balance between excitatory and suppressive features links to the balance theory of cortical circuits. The balance hypothesis is one of the leading theoretical frameworks within which cortical circuits and their computational functions are analyzed [36,37]. The quadratic filter was designed to capture filtering properties of local recurrent circuits. The fact that it turns out to have similar number of excitatory and suppressive features serves as supporting evidence for the balanced hypothesis in cortical circuits at the functional computational level. The fact that this balance was maintained at similar levels across different brain regions further speaks to the generality of balanced networks across different brain regions, which have so far been studied predominantly in the primary visual cortex.

For both neural and machine learning systems, the multiple stages of processing are generally believed to yield selectivity to more extended objects [38–40]. Despite exhibiting more invariance, real neurons maintain their selectivity across stages of processing [41]. The analyses described above show that quadratic computations contribute to the maintenance of this selectivity. Suppressive features are a key part of the quadratic computations. They can provide a mechanism not only for cross-orientation suppression mentioned above, but also for surround suppression [42] and contrast normalization more generally. Contrast normalization in particular is appreciated for allowing neurons to maintain information transmission across changes in stimulus conditions [43], through both adaptive [44,45] and fixed mechanisms of individual neurons (via nonlinear gain function implementing threshold and saturation) [46–48]. Suppressive features add to these nonlinear mechanisms by capturing the contributions of local recurrent circuits to contrast normalization. From this perspective, the increase in sparseness from the action of suppressive features is in the same vein as previous observations of increases of information transmission from contrast normalization [43]. After all, sparseness is closely related to the Shannon mutual information [49].

Notably, increased selectivity came not only from the suppressive computations, but also from the alignment in pooling across stages of processing (Fig 6F). This increase in response sparseness is unexpected because typically pooling across similarly tuned subunits would increase invariance and therefore decrease sparseness. However, because of nonlinearities within each subunit, the overall result is the increased refinement of stimulus selectivity (Fig 4C) and increase in response sparseness on average across a set of natural stimuli (Fig 6F).

Another unexpected observation here was the clear dominance of either the linear or quadratic computation at each model layer (Fig 2). We observed neurons that were linear at the first model layer, and quadratic at the second model layer, and vice versa, as well as those that were linear/quadratic in both model layers. One way to interpret this finding is to note that quadratic computations likely reflect cases with strong recurrent processing. Therefore, the observed dichotomy may reflect the parallel signaling pathways that connect brain areas via cortical connections [50] or thalamus [51]. Within our modeling framework, thalamic processing would contribute more linear computations. The observation of four classes of cells builds upon and expands the classic simple and complex receptive field mechanisms and suggests new processing stages to add to deep learning networks.

Overall, our results suggest how neurons respond to the interactions of multiple features in order to increase their selectivity for a specific type of stimulus. Within a computational layer, features congruent with these stimuli combine to increase the neuron’s response while those inconsistent with these stimuli are suppressed to sharpen the neural response. Across computational layers, pooling responses along the preferred orientation of the previous layer increases selectivity, responding more strongly when a feature extends beyond the receptive field of the first computational layer. Analysis of areas beyond V4 will likely require incorporating additional layers of the model to make it deeper. Nevertheless, we hope that the basic quadratic convolution motif introduced here will continue to generate improvements in predictive power and interpretability in higher brain areas as we have observed here for mid-level visual processing areas.

## Methods

### Electrophysiological recordings

We fit our model to neuronal recordings from four different datasets. The first dataset was from the primary visual cortex of cats [19]. Tetrode electrodes recorded spike trains as the anesthetized animals were exposed to natural movie stimuli. The movies were 128×128 pixels with a resolution of $0.12^{\circ }$ per pixel. We downsampled the movies to 32×32 pixels before fitting our model.

The second dataset was recorded from the primary visual cortex of macaque monkeys [52]. Tungsten electrodes recorded the spike trains while the animals fixated on the screen for juice rewards. The movies were natural image sequences or natural images with simulated saccades. The size of the movies was 50×50 pixels with a resolution of 0.1-$0.5^{\circ }$ per pixel. We took the central 40 pixels and downsampled them to 20×20 pixels before fitting.

The third dataset was recorded from visual area V2 of macaque monkeys [18]. The spike trains were recorded while the animals performed a fixation task for liquid reward. The stimuli were random patches of images chosen to favor high contrast and were scaled to match the size of the measured classical receptive field. We downsampled the stimuli to 20\time20 pixels.

The final dataset was recorded from visual area V4 in awake macaque monkeys [20]. The spikes were also recorded during a fixation task. The stimuli were natural movies displayed at a 120×120-pixel resolution with a fixed size of $14\times 14^{\circ }$. The movies were downsampled to 20×20 pixels and were analyzed in color.

### Low-rank quadratic convolutional model

The model seeks to make a prediction (${\hat{Y}}_{t}$) of a neuron’s response *Y*_*t*_ (measured in action potentials/spikes per time bin) given the stimulus ${𝐗}_{t}$ presented. The stimulus was normalized to have a mean of zero and a variance of one.

The stimulus begins with a size of $D_{t}^{\left(0\right)}$ × $D_{c}^{\left(0\right)}$ × $D_{x}^{\left(0\right)}$ × $D_{y}^{\left(0\right)}$, where *D* is the size along the time, color, horizontal, and vertical directions. In the first layer, the stimulus is convolved with ${\text{H}}^{\left(1\right)}$, ${\text{U}}^{\left(1\right)}$, and ${\text{V}}^{\left(1\right)}$. ${\text{H}}^{\left(1\right)}$ is the linear term with a single component. ${\text{U}}^{\left(1\right)}$ and ${\text{V}}^{\left(1\right)}$ combine to form the quadratic term and have ${\text{m}}^{\left(1\right)}$ components. Each component has size $1\times 1\times d^{\left(1\right)}\times d^{\left(1\right)}$. The outputs of these convolutions are

$${𝐡}^{\left(1\right)}=H^{\left(1\right)}*𝐗$$ (1)

$${𝐮}^{\left(1\right)}=U^{\left(1\right)}*𝐗$$ (2)

$${𝐯}^{\left(1\right)}=V^{\left(1\right)}*𝐗,$$ (3)

and they are combined like

$${𝐱}^{\left(1\right)}=a^{\left(1\right)}+{𝐡}^{\left(1\right)}+\sum_{j=1}^{m^{\left(1\right)}}{𝐮}_{j}^{\left(1\right)}{𝐯}_{j}^{\left(1\right)}.$$ (4)

${\text{a}}^{\left(1\right)}$ is a scalar bias. This is passed through a logistic function to give the output of the first layer:

$${𝐫}^{\left(1\right)}=\frac{1}{1+\exp(-{𝐱}^{\left(1\right)})}.$$ (5)

The second layer is similar to the first with the following exceptions: the kernels are dotted with the input rather than convolved, the components have size $D_{t}^{\left(0\right)}\times D_{c}^{\left(0\right)}\times d^{\left(2\right)}\times d^{\left(2\right)}$ where $d^{\left(2\right)}=D_{x}^{\left(0\right)}-d^{\left(1\right)}+1$, and the activation function is

$$\hat{Y}=c\log\left(1+\exp{𝐱}^{\left(2\right)}\right).$$ (6)

*c* is a positive scalar that adjusts the mean response. In total, the model has nine sets of parameters: ${\text{a}}^{\left(1\right)}$, ${𝐇}^{\left(1\right)}$, ${𝐔}^{\left(1\right)}$, ${𝐕}^{\left(1\right)}$, ${\text{a}}^{\left(2\right)}$, ${𝐇}^{\left(2\right)}$, ${𝐔}^{\left(2\right)}$, ${𝐕}^{\left(2\right)}$, and *c*.

### Fitting the model

The model was implemented using the Keras python package using Theano as a backend. We used the adadelta optimizer and terminated optimization when the Poisson log-likelihood on the validation set failed to improve for more than two passes.

The model has nine hyper parameters: the spatial size of the first layer kernels $\left({\text{d}}^{\left(1\right)}\right)$, the ranks of the quadratic filters for each layer $\left({\text{m}}^{\left(1\right)},{\text{m}}^{\left(2\right)}\right)$, individual regularization parameters for each layer’s linear and quadratic filters ($\lambda_{H^{\left(1\right)}}$, $\lambda_{H^{\left(2\right)}}$, $\lambda_{UV^{\left(1\right)}}$, $\lambda_{UV^{\left(2\right)}}$), and regularization parameters for the difference ${\text{UU}}^{\text{T}}$–${\text{VV}}^{\text{T}}$ for each layer ($\epsilon_{UV^{\left(1\right)}}$,$\epsilon_{UV^{\left(2\right)}}$). ${\text{d}}^{\left(1\right)}$ ranged from 6 to 18 (28 in the case of cat V1). ${\text{m}}^{\left(1\right)}$ ranged from 1 to 24, and ${\text{m}}^{\left(2\right)}$ ranged from 1 to 20. $\lambda_{H^{\left(1\right)}}$, $\lambda_{H^{\left(2\right)}}$, $\lambda_{UV^{\left(1\right)}}$, $\lambda_{UV^{\left(2\right)}}$, $\eta_{UV^{\left(1\right)}}$, and $\eta_{UV^{\left(2\right)}}$ all ranged from 10^−7^ to 10^0^, distributed logarithmically.

We chose the hyperparameters that produced the best predictions on a cross-validation set consisting of a quarter of the training/cross-validation data. We fit a model to each of four different training/cross-validation splits and used the average correlation with the observed responses as our performance metric. In order to choose the values of the hyperparameters, we used the Spearmint package [53], which fits the performance in the space of hyperparameters as a Gaussian process. Based on this fit, the program suggests new values of the hyperparameters that have the highest expected improvement. We ran 100 sets of hyperparameters and selected the set with the best correlation for further analysis.

This optimization method allowed us to manage the trade-off between the increased explanatory potential of a larger number of parameters with the danger of overfitting. This is primarily handled by the hyperparameters ${\text{m}}^{\left(1\right)}$ and ${\text{m}}^{\left(2\right)}$ which control the rank of the first and second layers’ quadratic filters and therefore the number of parameters each has. This trade-off increasingly favored the number of parameters for later areas, starting with a median spikes per parameter of 13 and 8 for cat V1 and macaque V1 and going down to 3 and 2 for macaque V2 and macaque V4, respectively.

### Benchmark model

In order to determine how well our model was at predicting neural responses, we also fit XGBoost models [21].

### Evaluating model performance

To calculate the performance of the models, we evaluated

$$acosh\left(\frac{1}{\rho^{2}}\right)$$ (7)

for subsets of the test dataset of varying lengths (where ρ was the correlation) and calculated a linear regression with respect the inverse of the number of samples in the subset [54,55]. The y-intercept of this line corresponds to the projected value in the limit of infinite data. This procedure aims to remove bias due to coincidental correlations. Inverting Eq 7 provides our estimate of the correlation between our model predictions and the observed responses.

### Dividing cells into linear and quadratic classes

We calculated the variances of the linear (${𝐱}_{𝐡}$) and quadratic ($\sum {𝐱}_{U_{i}}{𝐱}_{V_{i}}$) contributions to each later to get $\sigma_{{𝐡}^{\left(1\right)}}^{2}$, $\sigma_{UV^{\left(1\right)}}^{2}$, $\sigma_{{𝐡}^{\left(2\right)}}^{2}$, and $\sigma_{UV^{\left(2\right)}}^{2}$. With these we calculated the fraction of the layer’s variance contributed by the quadratic component

$$QuadraticIndex=\frac{\sigma_{UV^{\left(i\right)}}^{2}}{\sigma_{UV^{\left(i\right)}}^{2}+\sigma_{{𝐡}^{\left(i\right)}}^{2}}.$$ (8)

If the quadratic index was greater than 0.5, we classified the layer as quadratic (Q1 for the first layer and Q2 for the second). Otherwise, we classified it as linear (L1 or L2). Each layer was classified independently, creating four classes: Linear-Linear (LL), Linear-Quadratic (LQ), Quadratic-Linear (QL), and Quadratic-Quadratic (QQ).

### Feature fitting using differential evolution

In order to extract perceptually relevant properties from the parameter weights of the model, we fit the kernels as various types of features using differential evolution [56], which randomly initializes sets of parameters and generates new sets by taking a set and modifying random values using the values of the other sets. If the child set has a lower error than its parent, it takes its parent’s place. The specific variation of the algorithm is termed “rand/2/bin” [1].

The types of features that we used to analyze each neuron depended on what class (LL, QL, LQ, QQ) the neuron belonged to. For neurons dominated by the quadratic term on the first layer, we fit the quadratic kernel ${\text{J}}^{\left(1\right)}$ using curved Gabor wavelets:

$$𝐠=\exp\left(-\frac{{𝐮}^{2}+{𝐯}^{2}\gamma^{2}}{2\sigma^{2}}\right)\cos\left(\frac{2\pi }{\lambda \sigma }𝐮+\phi \right)$$ (9)

$$J_{CG}^{\left(1\right)}=\sum_{i}w_{i}{𝐠}_{i}{𝐠}_{i}^{T},$$ (10)

where


$$(\begin{array}{c} {𝐱}^{\prime }\\ {𝐲}^{\prime } \end{array})=(\begin{array}{c} 𝐱-x_{0}\\ 𝐲-y_{0}\; \end{array})R_{\theta }$$

and


$$𝐮={𝐱}^{\prime }-\frac{\kappa }{\sigma }{𝐲}^{\prime 2}$$

$$𝐯={𝐲}^{\prime }.$$

The parameters for each curved Gabor wavelet are the weight (*w*), the position ($x_{0},y_{0}$), the orientation (θ), the curvature (κ), the aspect ratio (γ), the size (σ), the spatial wavelength (λ), and the phase (ϕ).

Variations were also tested. Uncurved models fixed κ to be zero while paired models had pairs of Gabor wavelets with identical parameters except that ϕ was set to be 0 and π/2. The combination of paired and unpaired, curved and uncurved created four alternative models.

For the second layer quadratic cells, we fit two moving cosine functions:

$$𝐠=\cos\left(\frac{2\pi }{\lambda }{𝐱}^{\prime }+\omega t+\phi \right).$$ (11)

Fig 1A shows the structure of the model. The stimulus has two-spatial dimensions, a color dimension (in the case of V4 data), and a temporal dimension. A series of linear spatial kernels are convolved across each position, time, and color (if applicable). One of these kernels is treated linearly while the rest are passed through a quadratic function before being combined into a single value at each part of the stimulus. These values are passed through a sigmoid function to create the input for the second layer. Another set of linear and quadratic kernels are applied to this input to create a single value that is passed through a rectifying nonlinearity to produce a predicted firing rate.

### Local orientation difference

Starting with the parameters of a curved Gabor, one can calculate the local orientation using

$$\psi (x,y)=\arctan\left(\frac{\sin\theta +2\kappa \cos\theta ((y-y_{0})\cos\theta +(x-x_{0})\sin\theta )}{\cos\theta -2\kappa \sin\theta ((y-y_{0})\cos\theta +(x-x_{0})\sin\theta )}\right).$$ (12)

Each local orientation was given a weight with three factors: |w| (the Gabor’s weighting in *J*), the weight of the curved Gaussian envelope *a*(*x*,*y*), and the norm of the projection of the Gabor into the significant eigenvectors

$$b={(\sum_{j=1}^{n}(𝐠\cdot {\hat{e}}_{j}{)}^{2})}^{0.5}.$$ (13)

Using the weighted angular mean

$$\text{anglemean}\left(\alpha ,𝐰\right)=\text{angle}\left(\sum_{j}\exp(i\alpha_{j})w_{i}\right),$$ (14)

we calculated the mean excitatory or suppressive orientation at a location as

anglemean(2ψ(x,y),𝐰𝐚(x,y)𝐛)/2. (15)

The factors of two causes ψ and ψ  +  π to add together rather than cancel each other out since the orientation is not directional.

We selected the point of maximum interaction between excitation and suppression by summing the weights at each location for the excitatory and suppressive Gabors respectively and then multiplied them together. Using the absolute angular difference

$$\Delta \theta (\theta_{1},\theta_{2})=\pi -|\pi -\;\mathrm{mod}\;\left(|\theta_{1}-\theta_{2}|,2\pi \right)|,$$ (16)

the difference between the excitatory and suppressive orientation preference is

$$\Delta \theta (2\psi_{E}(x_{max},y_{max}),2\psi_{S}(x_{max},y_{max}))/2.$$ (17)

Once again, the factor of two makes the calculation direction independent.

### Measuring orientation and direction selectivity in second-layer features

Given a spatiotemporal feature *a*_*x*,*y*,*t*_, we calculated the dominant orientation by first calculating the Fourier transform with respect to the two spatial dimensions: ${\hat{a}}_{l,m,t}$. We also calculated the orientation of each Fourier component

$$k_{x}=\;\mathrm{mod}\;\left(\frac{2\pi l}{N_{x}}+\frac{\pi }{2},\pi \right)-\frac{\pi }{2}$$ (18)

$$k_{y}=\;\mathrm{mod}\;\left(\frac{2\pi m}{N_{y}}+\frac{\pi }{2},\pi \right)-\frac{\pi }{2}$$ (19)

$$\theta_{l,m}=\text{angle}\left(k_{x}+k_{y}i\right).$$ (20)

These angles are averaged together weighted by the Fourier transform

$$\bar{\theta }=\text{anglemean}\left(2\theta_{l,m},|{\hat{a}}_{l,m,t}|\right)/2.$$ (21)

The weights for the zero spatial frequency components and the Nyquist frequency components are set to zero since the orientation is undefined. The factors of two ensures that θ and θ+π are treated as the same orientation.

Measuring the preferred direction is done similarly with a few modifications. The Fourier transform is taken with respect to the temporal dimension in addition to the spatial dimensions to produce ${\hat{a}}_{l,m,n}$. The angle is also calculated slightly differently:

$$\omega_{t}=\;\mathrm{mod}\;\left(\frac{2\pi n}{N_{t}}+\frac{\pi }{2},\pi \right)-\frac{\pi }{2}$$ (22)

$$\theta_{l,m,n}=\text{angle}\left(\omega_{t}*(k_{x}+k_{y}i)\right).$$ (23)

The angles are averaged using

$$\bar{\theta }=\text{anglemean}\left(\theta_{l,m,n},|{\hat{a}}_{l,m,n}|\right).$$ (24)

In addition to the non-oriented components zeroed above, the components for $\omega_{t}=0$ were also zeroed as they had no directed motion. The factors of two are removed from this variation because θ and θ+π are no longer equivalent.

The moving grating that found the maximum (minimum) response was found by using differential evolution to generate gratings of the form

$$\cos\left(\omega t+k_{x}x+k_{y}y+\phi \right).$$ (25)

For the macaque V4, the color was determined by adding three additional parameters representing the color in HSV space. This color was converted to RGB and then normalized to unit length. The algorithm selected the candidates with the higher (lower) response from the model until convergence.

To measure direction selectivity, *k*_*x*_ and *k*_*y*_ were replaced with

$$k_{x}(\theta )=k\cos(\theta )$$ (26)

$$k_{y}(\theta )=k\sin(\theta ),$$ (27)

where $k=(k_{x}^{2}+k_{y}^{2}{)}^{0.5}$ and θ ranged from 0 to 2π. The directional circular variance was calculated from the responses:

$$L_{dir}=\frac{\left|\sum_{j}R(\theta_{j})\exp(i\theta_{j})\right|}{\sum_{j}R(\theta_{j})}.$$ (28)

The circular variance ranges from 0 when the response is uniform with respect to θ to 1 when only a single direction provokes a response.

## Supporting information

S1 Fig Individual curved Gabor have better AIC scores than alternative models for all areas. Quadratic and linear comparisons only for those cells that were quadratic or linear in the first layer, respectively. Examples of the alternative Gabor models are below their respective bars with the dominant curved Gabor models on the top.
(PDF)

S2 Fig V4 cells are more selective for motion. The dominant filter of the second layer of V4 neurons had a higher motion selectivity index than the other areas we studied, i.e. the filters were less separable into spatial and temporal components. This indicates that the models for V4 had a higher capacity to select for motion. This may be in part due to the differences in the stimuli used in the experiments.
(PDF)

S3 FigOrientation distribution at the first layer biases inputs to the second layer towards its orientationDistribution of oriented energy in Gaussian noise (black), natural movies (green), the first layer with a linear filter (red), and the first layer with a pair of excitatory features in the quadratic filter. The insets show examples of the associated images. The distributions for horizontal, diagonal ($45^{\circ }$), and vertical features are shown. The linear example created the largest shift in the distribution relative to the natural image inputs, but the quadratic example also shifted the distribution towards the preferred direction of the first layer’s features.(PDF)

S4 Fig Number of relevant features per neuron at each layer of the model. A, B. The number of features in the first features for the quadratic cells only or for all cells counting the linear cells as having one dimension. For both measures, V4 has more features than earlier visual areas. **C, D.** The number of features for the second layer. The number of features was not significantly different across areas except that cat V1 had significantly fewer when linear cells were included.
(PDF)

S5 Fig Balance of excitation and suppression across brain areas. A. The distribution of the relative strength of excitation in the first layer as a fraction of the total eigenvalue weights. There were no significant differences between the areas. **B.** The excitatory fraction for the second layer. Macaque V1 had more suppression than the other areas.
(PDF)

S6 Fig Sparseness scatter plot for cat V1. Scatter plot of data in Fig 6.
(PDF)

S7 Fig Sparseness scatter plot for macaque V1. Scatter plot of data in Fig 6.
(PDF)

S8 Fig Sparseness scatter plot for macaque V2. Scatter plot of data in Fig 6.
(PDF)

S9 Fig Sparseness scatter plot for macaque V4. Scatter plot of data in Fig 6.
(PDF)

S10 FigStimulus statistics limit types of computations that can be observed. A.Fourier spectrum along the temporal and horizontal axes. The simulated saccade stimulus (used for macaque V1) had almost all of its power in the stationary band. The natural movie stimulus (used for cat V1 and macaque V4) has power spread through the motion bands, decaying in intensity with increasing spatial and temporal frequency. The image sequence stimulus (use for macaque V2) has a lot of power spread evenly across different temporal frequencies with decaying intensity with increasing spatial frequency. All frequencies are in fractions of π radians. **B.** The response of different models to sine grating stimuli. We designed a base model with strong selectivity for motion of a particular spatial and temporal frequency to test whether models trained from spikes generated from the various stimuli could recover this behavior. The models trained on responses generated from simulated saccade and image sequence stimuli were not able to reproduce the motion selectivity, finding only the weaker selectivity to stationary stimuli. In contrast, the natural movie stimulus was able to respond strongly to the motion preferred by the base model. This is likely due to the natural movies having plenty of examples of coherent motion. The simulated saccade stimuli had extremely limited representations of any motion, and the image sequence had a lot of motion, but it is unlikely that a sequence of five images would have the phases of their spatial frequencies coordinate in a way to give the impression of constant motion.(PDF)

S11 Fig Moving gratings that maximize and minimize response for example neuron. For the example neuron shown in Fig 5, the moving sine gratings that maximize and minimize the response for a given intensity. As predicted from inspection of the first layer’s linear filter and eigenvectors of the second layer’s quadratic filter, the neuron responds most strongly to gratings moving to the up and right and least strongly to gratings moving down and to the left.
(PDF)

S12 Fig Models with RELU activation function in first layer show more mixed contributions from linear and quadratic filters. While the model quadratic index in each combination of dataset and layer still has either the linear (index of 0) or quadratic (index of 1) filter dominating the layer’s response, many more models had intermediate values of the quadratic index compared to models fit with a sigmoid function, except in cat V1.
(PDF)

S13 Fig Direction selectivity found in cells from all areas. The distribution of the directional circular variance of the responses of models to optimal sine gratings with different orientations. While the models for most cells show low directional selectivity, direction selective cells can be found in all areas.
(PDF)
