# Supplementary material for: Computations that sustain neural feature selectivity across processing stages
Source: PLoS Comput Biol. 2025 Jun 20;21(6):e1013075. doi: 10.1371/journal.pcbi.1013075 (PMC12180665; doi:10.1371/journal.pcbi.1013075)
Supplement: S1 Fig — Quadratic and linear comparisons only for those cells that were quadratic or linear in the first layer, respectively. Examples of the alternative Gabor models are below their respective bars with the dominant curved Gabor models on the top. (PDF) [file pcbi.1013075.s001.pdf]

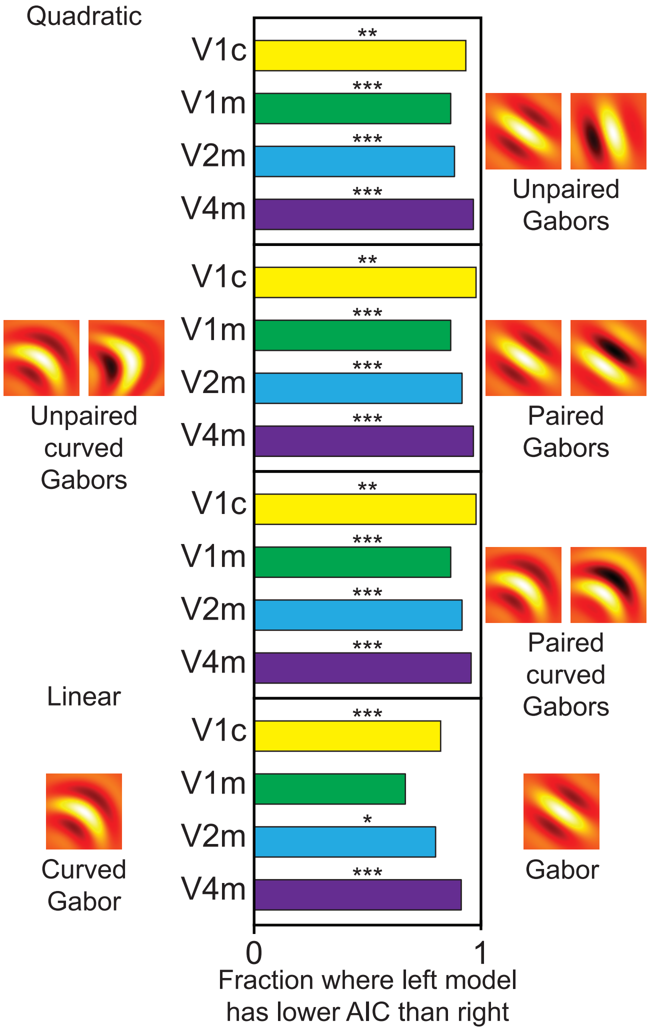
Quadratic
Linear
V1c
V1m
V2m
V4m
**
***
*
Unpaired Gabors
Paired Gabors
Paired curved Gabors
Gabor
Unpaired curved Gabors
Curved Gabor
0
1
Fraction where left model has lower AIC than right
