# Supplementary figures and images for: Computations that sustain neural feature selectivity across processing stages
Source: PLoS Comput Biol. 2025 Jun 20;21(6):e1013075. doi: 10.1371/journal.pcbi.1013075 (PMC12180665; doi:10.1371/journal.pcbi.1013075)

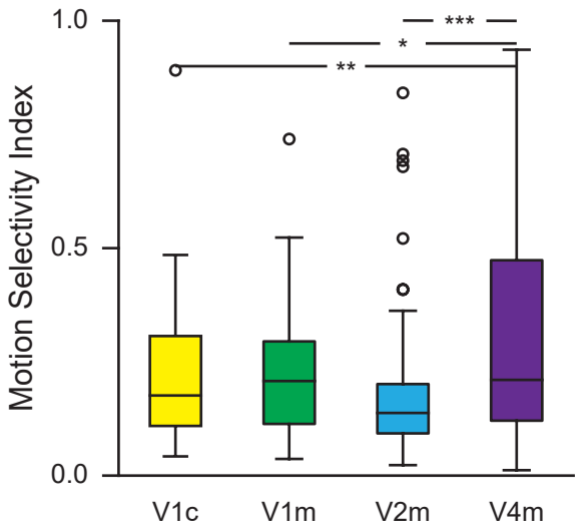

Supplement: S2 Fig — The dominant filter of the second layer of V4 neurons had a higher motion selectivity index than the other areas we studied, i.e. the filters were less separable into spatial and temporal components. This indicates that the models for V4 had a higher capacity to select for motion. This may be in part due to the differences in the stimuli used in the experiments. (PDF) [file pcbi.1013075.s002.pdf]

Horizontal

90°

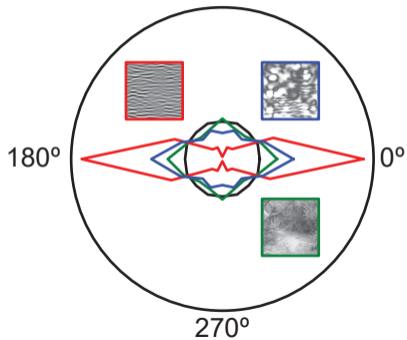

Diagonal

90°

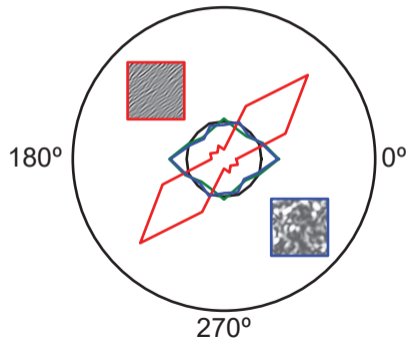

Vertical

90°

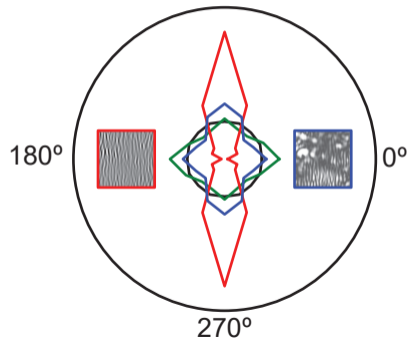

Supplement: S3 Fig — Distribution of oriented energy in Gaussian noise (black), natural movies (green), the first layer with a linear filter (red), and the first layer with a pair of excitatory features in the quadratic filter. The insets show examples of the associated images. The distributions for horizontal, diagonal (45∘), and vertical features are shown. The linear example created the largest shift in the distribution relative to the natural image inputs, but the quadratic example also shifted the distribution towards the preferred direction of the first layer’s features. (PDF) [file pcbi.1013075.s003.pdf]

# Layer1

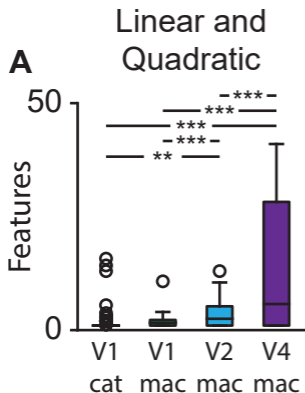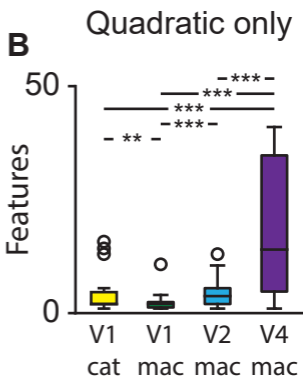

# Layer 2

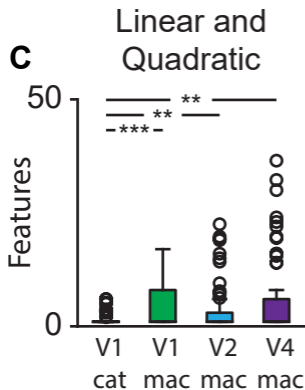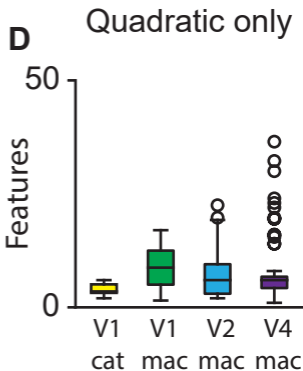

Supplement: S4 Fig — The number of features in the first features for the quadratic cells only or for all cells counting the linear cells as having one dimension. For both measures, V4 has more features than earlier visual areas. C, D. The number of features for the second layer. The number of features was not significantly different across areas except that cat V1 had significantly fewer when linear cells were included. (PDF) [file pcbi.1013075.s004.pdf]

**A**

Layer 1

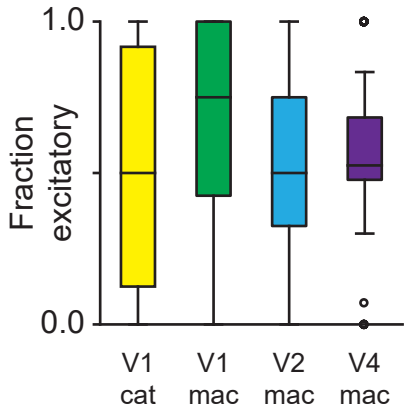**B**

Layer 2

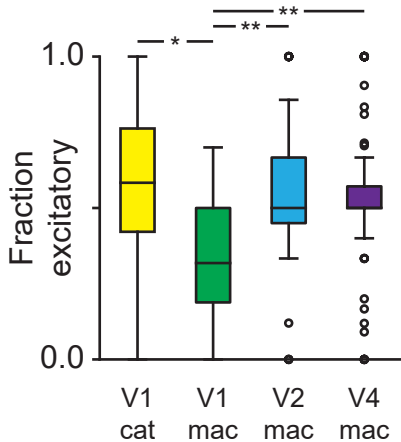

Supplement: S5 Fig — The distribution of the relative strength of excitation in the first layer as a fraction of the total eigenvalue weights. There were no significant differences between the areas. B. The excitatory fraction for the second layer. Macaque V1 had more suppression than the other areas. (PDF) [file pcbi.1013075.s005.pdf]

# Sparseness - Cat V1

**A**

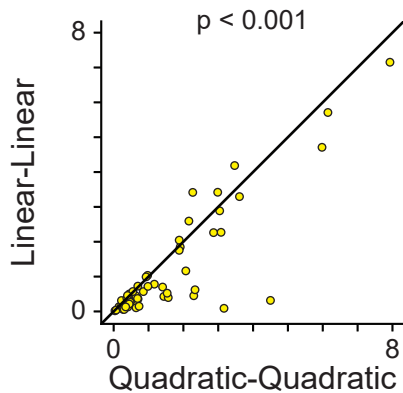

**B**

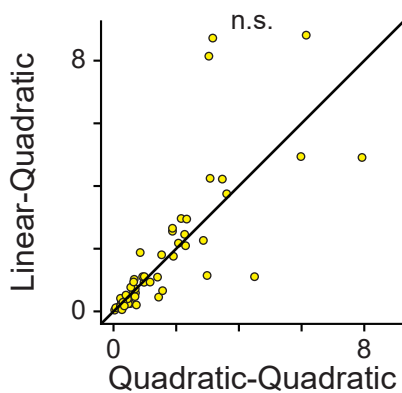

**C**

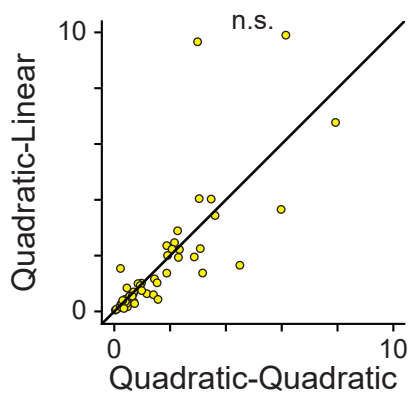

Base model versus rotating (left):

**D**

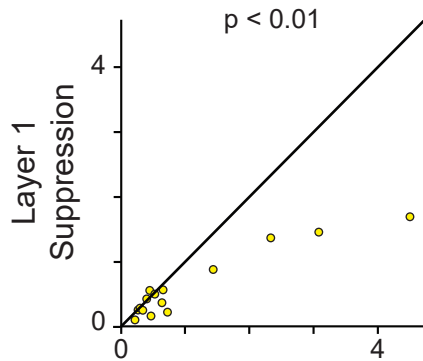

**E**

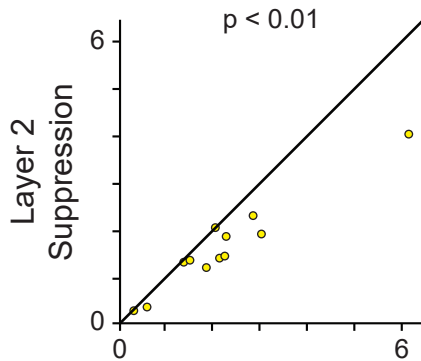

**F**

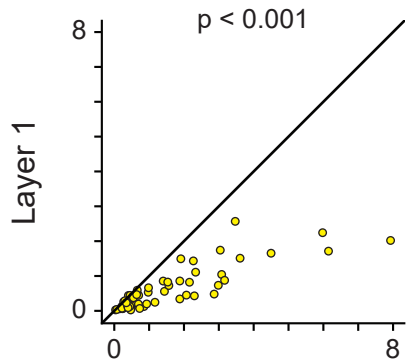

Supplement: S6 Fig — Scatter plot of data in Fig 6. (PDF) [file pcbi.1013075.s006.pdf]

# Sparseness - Macaque V1

**A**

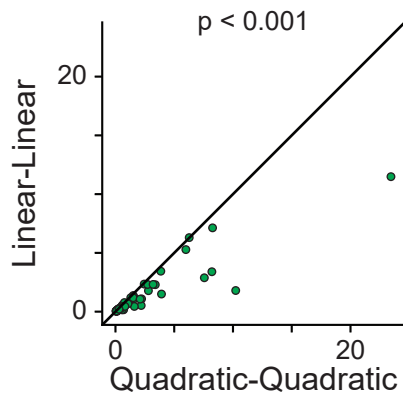

**B**

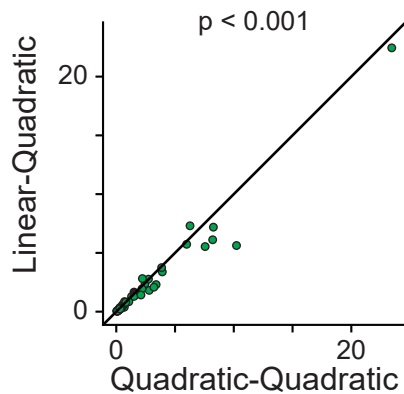

**C**

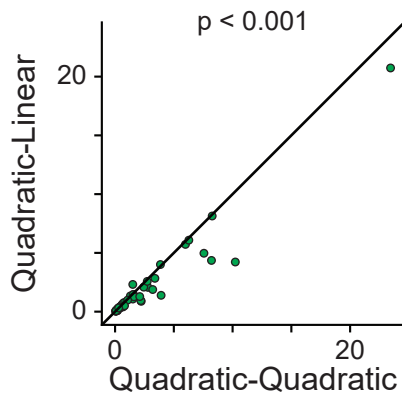

Base model versus rotating (left):

**D**

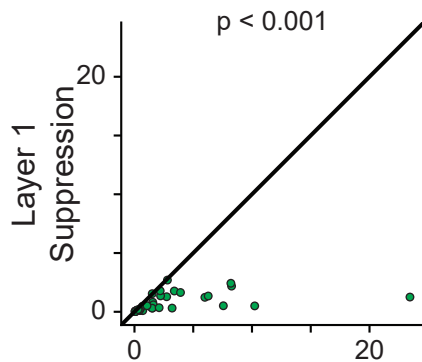

**E**

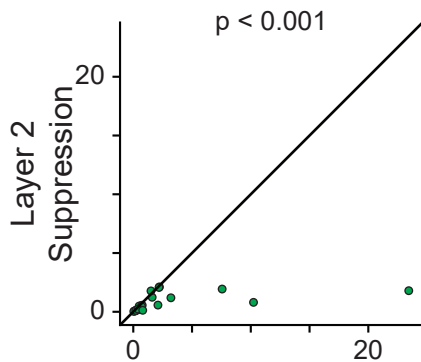

**F**

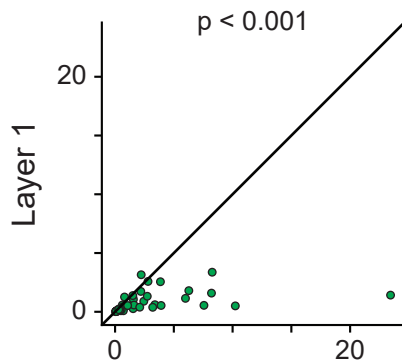

Supplement: S7 Fig — Scatter plot of data in Fig 6. (PDF) [file pcbi.1013075.s007.pdf]

# Sparseness - Macaque V2

**A**

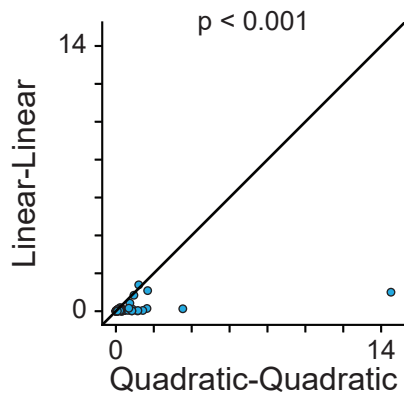

**B**

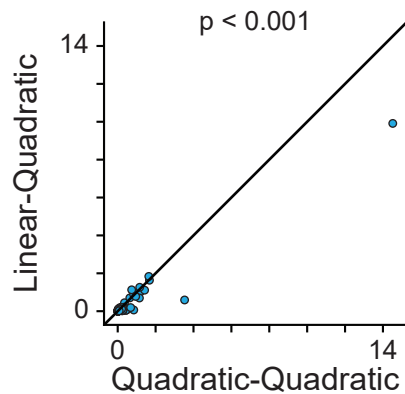

**C**

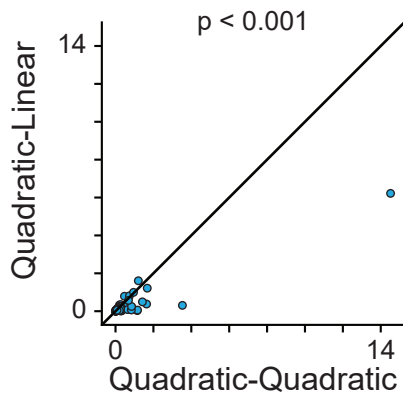

Base model versus rotating (left):

**D**

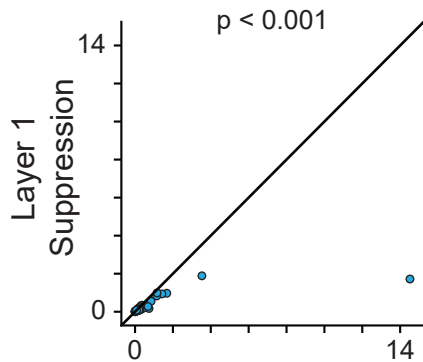

**E**

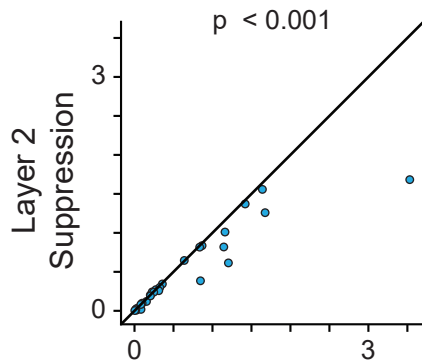

**F**

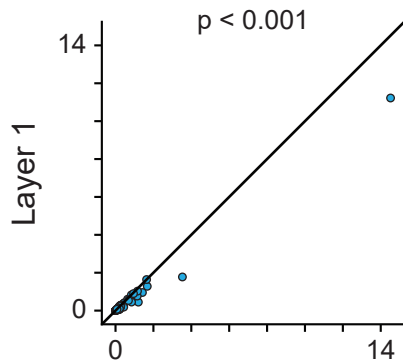

Supplement: S8 Fig — Scatter plot of data in Fig 6. (PDF) [file pcbi.1013075.s008.pdf]

# Sparseness - Macaque V4

**A**

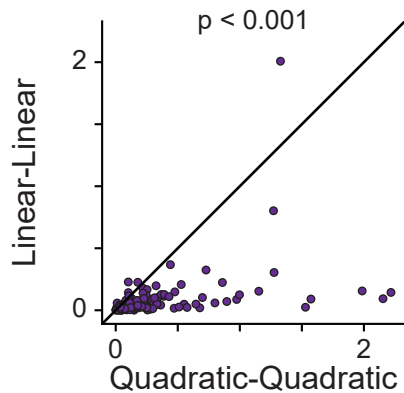

**B**

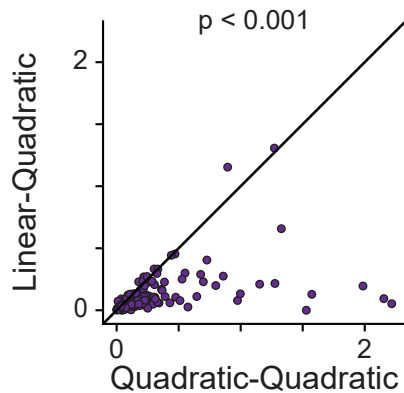

**C**

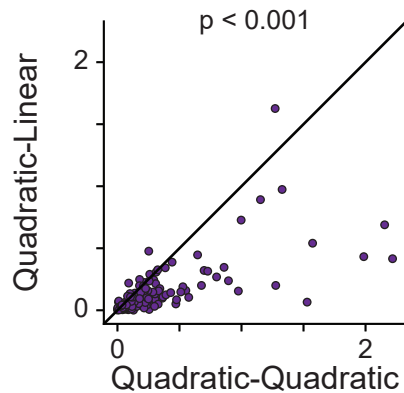

Base model versus rotating (left):

**D**

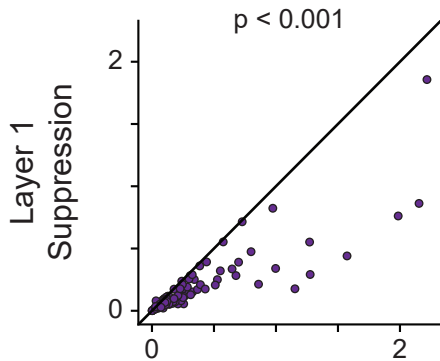

**E**

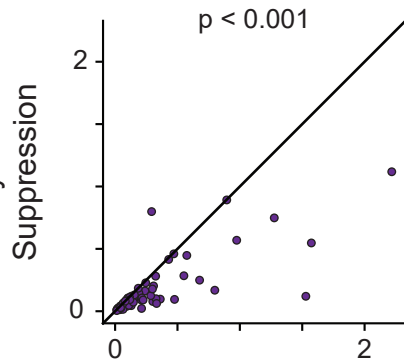

**F**

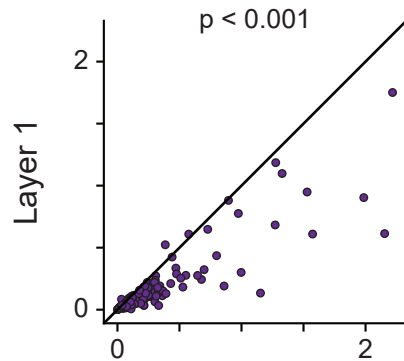

Supplement: S9 Fig — Scatter plot of data in Fig 6. (PDF) [file pcbi.1013075.s009.pdf]

## A Stimulus statistics

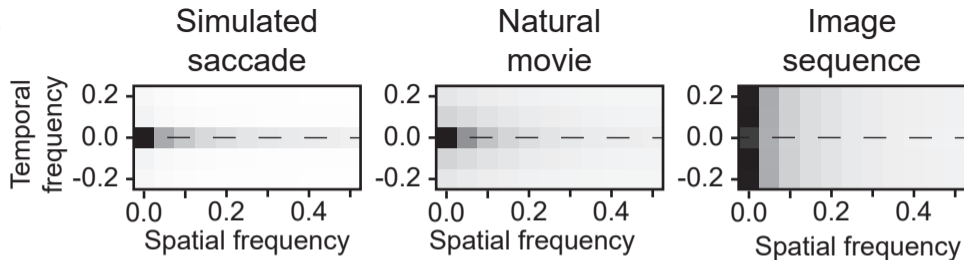

## B Model response to grating

Model fit to spikes generated with:

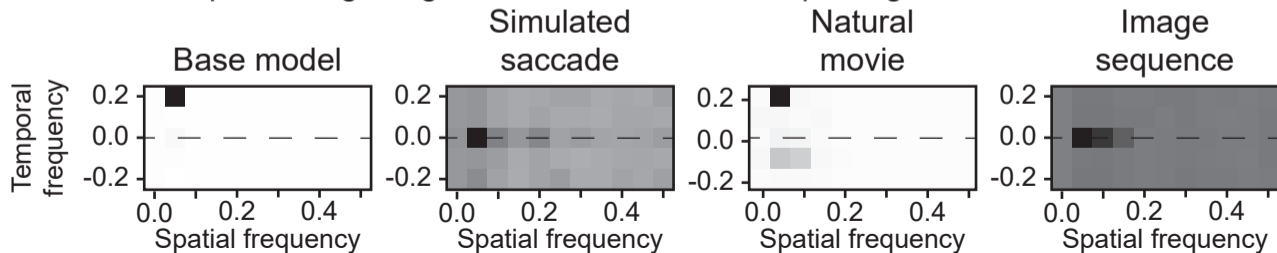

Supplement: S10 Fig — Fourier spectrum along the temporal and horizontal axes. The simulated saccade stimulus (used for macaque V1) had almost all of its power in the stationary band. The natural movie stimulus (used for cat V1 and macaque V4) has power spread through the motion bands, decaying in intensity with increasing spatial and temporal frequency. The image sequence stimulus (use for macaque V2) has a lot of power spread evenly across different temporal frequencies with decaying intensity with increasing spatial frequency. All frequencies are in fractions of π radians. B. The response of different models to sine grating stimuli. We designed a base model with strong selectivity for motion of a particular spatial and temporal frequency to test whether models trained from spikes generated from the various stimuli could recover this behavior. The models trained on responses generated from simulated saccade and image sequence stimuli were not able to reproduce the motion selectivity, finding only the weaker selectivity to stationary stimuli. In contrast, the natural movie stimulus was able to respond strongly to the motion preferred by the base model. This is likely due to the natural movies having plenty of examples of coherent motion. The simulated saccade stimuli had extremely limited representations of any motion, and the image sequence had a lot of motion, but it is unlikely that a sequence of five images would have the phases of their spatial frequencies coordinate in a way to give the impression of constant motion. (PDF) [file pcbi.1013075.s010.pdf]

## Response maximizing grating

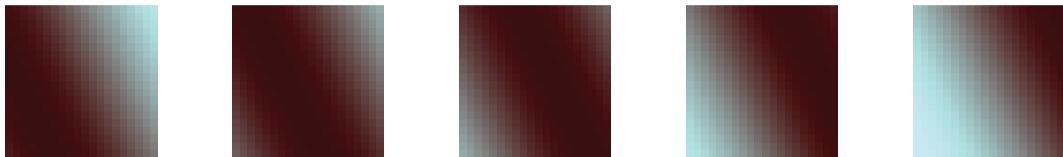

Time before  
spike (ms)

300

240

180

120

60

0

## Response minimizing grating

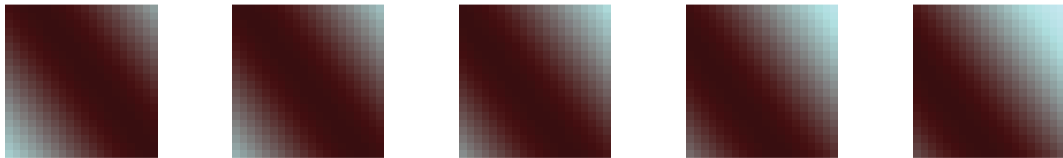

Supplement: S11 Fig — For the example neuron shown in Fig 5, the moving sine gratings that maximize and minimize the response for a given intensity. As predicted from inspection of the first layer’s linear filter and eigenvectors of the second layer’s quadratic filter, the neuron responds most strongly to gratings moving to the up and right and least strongly to gratings moving down and to the left. (PDF) [file pcbi.1013075.s011.pdf]

# Quadratic Index with RELU activation in Layer 1

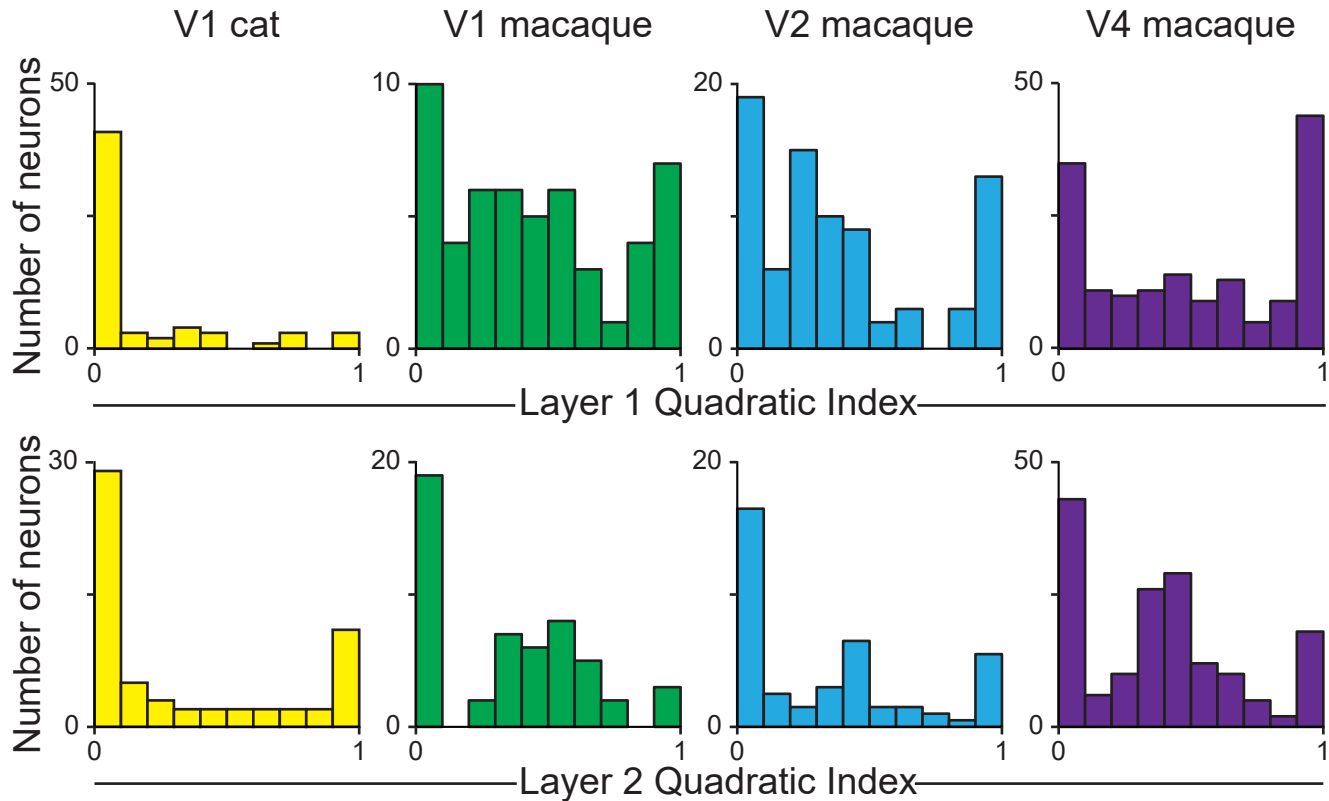

Supplement: S12 Fig — While the model quadratic index in each combination of dataset and layer still has either the linear (index of 0) or quadratic (index of 1) filter dominating the layer’s response, many more models had intermediate values of the quadratic index compared to models fit with a sigmoid function, except in cat V1. (PDF) [file pcbi.1013075.s012.pdf]

Number of neurons

V1 cat

V1 macaque

V2 macaque

V4 macaque

40

20

50

50

0

0

0

0

1

1

1

1

Circular Variance

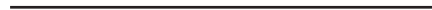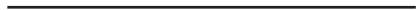

Supplement: S13 Fig — The distribution of the directional circular variance of the responses of models to optimal sine gratings with different orientations. While the models for most cells show low directional selectivity, direction selective cells can be found in all areas. (PDF) [file pcbi.1013075.s013.pdf]
